# A non-radioactive method for measuring Rubisco activase activity in the presence of variable ATP: ADP ratios, including modifications for measuring the activity and activation state of Rubisco

**DOI:** 10.1007/s11120-013-9964-5

**Published:** 2014-01-05

**Authors:** Joanna C. Scales, Martin A. J. Parry, Michael E. Salvucci

**Affiliations:** 1 Plant Biology and Crop Science, Rothamsted Research, Harpenden, Herts AL5 2JQ UK; 2 Arid-Land Agricultural Research Center, U.S. Department of Agriculture-Agricultural Research Service, 21881 N. Cardon Lane, Maricopa, AZ 85138 USA

**Keywords:** Carbon metabolism, Enzyme regulation, Molecular chaperone, AAA+

## Abstract

Rubisco (ribulose-1,5-bisphosphate carboxylase/oxygenase) catalyzes carboxylation of ribulose-1,5-bisphosphate, the first in a series of reactions leading to the incorporation of atmospheric CO_2_ into biomass. Rubisco requires Rubisco activase (RCA), an AAA+ ATPase that reactivates Rubisco by remodelling the conformation of inhibitor-bound sites. RCA is regulated by the ratio of ADP:ATP, with the precise response potentiated by redox regulation of the alpha-isoform. Measuring the effects of ADP on the activation of Rubisco by RCA using the well-established photometric assay is problematic because of the adenine nucleotide requirement of 3-phosphoglycerate (3-PGA) kinase. Described here is a novel assay for measuring RCA activity in the presence of variable ratios of ADP:ATP. The assay couples the formation of 3-PGA from ribulose 1,5-bisphosphate and CO_2_ to NADH oxidation through cofactor-dependent phosphoglycerate mutase, enolase, PEP carboxylase and malate dehydrogenase. The assay was used to determine the effects of Rubisco and RCA concentration and ADP:ATP ratio on RCA activity, and to measure the activation of a modified Rubisco by RCA. Variations of the basic assay were used to measure the activation state of Rubisco in leaf extracts and the activity of purified Rubisco. The assay can be automated for high-throughput processing by conducting the reactions in two stages.

**Electronic supplementary material:**

The online version of this article (doi:10.1007/s11120-013-9964-5) contains supplementary material, which is available to authorized users.

## Introduction

Improving the catalytic or regulatory properties of Rubisco to increase the rate of carbon assimilation in photosynthesis has been suggested as a strategy for boosting crop yields (Parry et al. 2013). Increasing the turnover rate of Rubisco or its affinity and/or specificity for CO_2_ (Spreitzer and Salvucci 2002; Whitney et al. 2011), preventing inactivation of Rubisco during periods of high temperature (Kurek et al. 2007; Parry et al. 2011; Carmo-Silva and Salvucci 2012) or improving the response time of Rubisco activation during transitions in light intensity (Carmo-Silva and Salvucci 2013) have all been proposed as possible ways to enhance the carbon fixing step of photosynthesis. To determine the effects of naturally-occurring or artificially-introduced modifications of Rubisco on carboxylation activity or the interaction with the catalytic chaperone, Rubisco activase (RCA), it is important to have a reliable method for measuring Rubisco and RCA activity. Ideally, the assay should be amenable to high throughput measurement of activity in plant tissue and with purified proteins. Given the central role of RCA in controlling the activation state of Rubisco, it is also desirable that the assay can measure RCA activity in response to variable ratios of ADP:ATP. The ratio of these adenine nucleotides is the major physiological factor affecting RCA activity (Robinson and Portis 1989a; Carmo-Silva and Salvucci 2013).

The activities of Rubisco and RCA are commonly measured by determining the rate of incorporation of ^14^CO_2_ into acid stable compounds using a short, timed assay (Lorimer et al. 1977). However, ^14^C is a hazardous material that requires safety precautions in its handling. This feature limits the use of the ^14^C-based assay to individuals with specialised training in the safe handling of radioactive material and liquid scintillation cocktail. Even with the proper training, the costs associated with a license to purchase, use and dispose of radioactive material, and to purchase and maintain a liquid scintillation counter can be prohibitive.

Photometric assays, either continuous (Sharkey et al. 1991) or two stage using enzyme cycling (Sulpice et al. 2007), offer alternative methods for measuring Rubisco activity. RCA activity can be measured by its ability to increase the activity of Rubisco and a continuous photometric assay for Rubisco has been adapted for use in measuring RCA activity (Lan et al. 1992; Esau et al. 1996). However, these assays employ 3-PGA kinase for the conversion of 3-PGA and ATP to 1,3-bisPGA. This enzyme exhibits a low affinity for ATP and a very high affinity for inhibition by ADP (Pacold and Anderson 1975). These properties preclude assay of RCA activity at variable ratios of ADP:ATP. This limitation is a drawback in the study of RCA because the sensitivity of RCA activity to inhibition by ADP is a major regulatory process controlling the activation state of Rubisco in response to irradiance and probably other environmental factors (Carmo-Silva and Salvucci 2013).

**Fig. 1:**
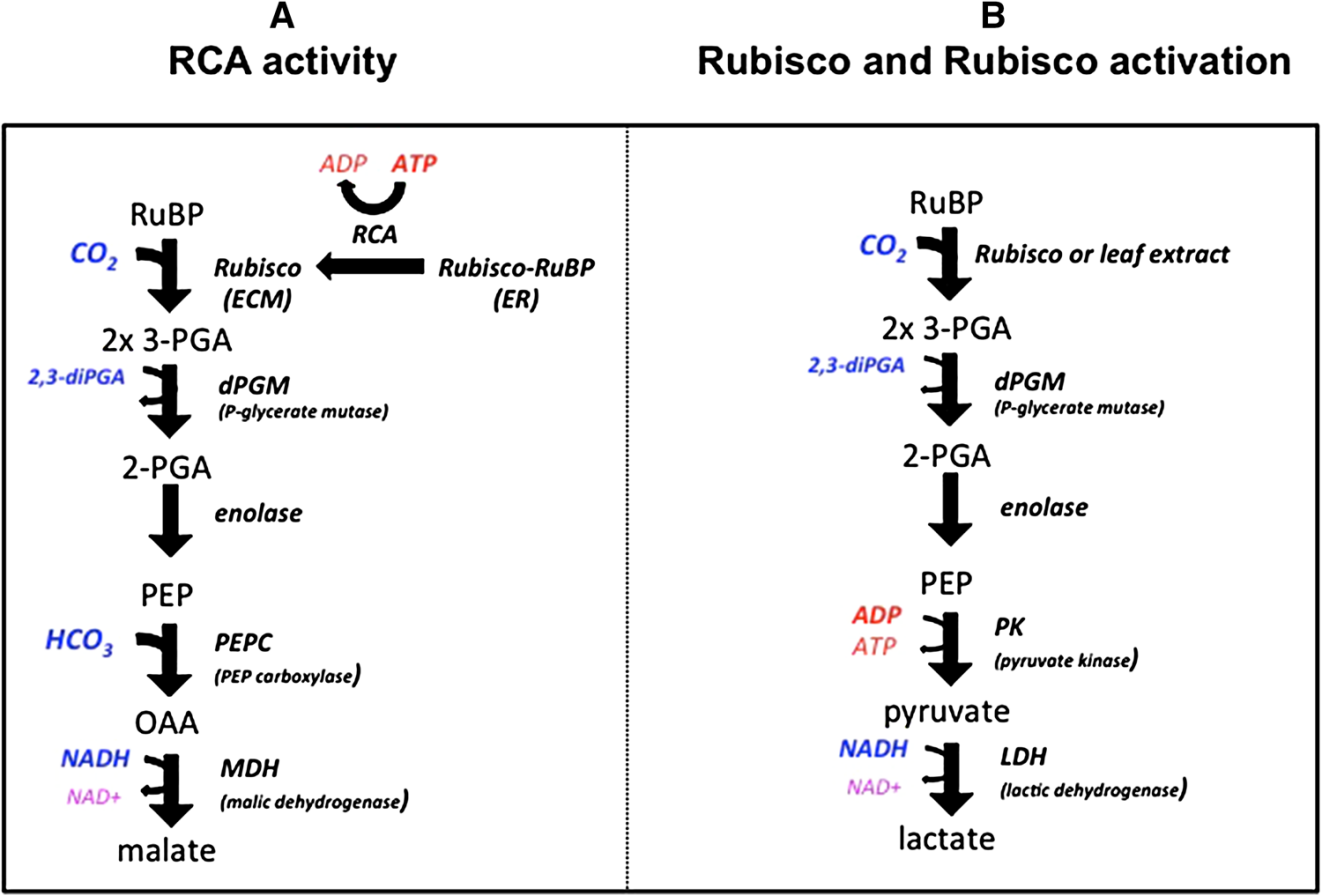
Reaction schemes for measuring the activities of RCA and Rubisco in continuous assays. The two diagrams show alternative pathways for coupling 3-PGA formation to NADH oxidation. **a** Pathway for measuring RCA activity. The coupling of 3-PGA formation to NADH oxidation is independent of adenine nucleotides, allowing measurement of RCA activity at variable ratios of ADP:ATP. **b** Pathway for measuring Rubisco and Rubisco activation. The coupling of 3-PGA formation to NADH oxidation requires ADP

A novel method for measuring Rubisco and RCA activity is described here. Instead of coupling 3-PGA formation to NADH oxidation via 3-PGA kinase, 2,3-bisPGA-dependent phosphoglycerate mutase (dPGM) was used to convert 3-PGA to 2-PGA (Fig. 1). Enolase was then used to convert 2-PGA to PEP. For measurement of RCA activity in the presence of variable ratios of ADP:ATP, the formation of PEP was coupled to NADH oxidation via PEP carboxylase and malic dehydrogenase. A modification of the basic method is described for the routine assay of Rubisco activity and Rubisco activation state. This modification replaces the PEP carboxylase–malate dehydrogenase link with pyruvate kinase and lactate dehydrogenase, two relatively inexpensive linking enzymes. By dividing the reaction into two stages, both the standard and the modified assays can be automated for high-throughput processing.

## Materials and methods

### Materials

Mention of a trademark, proprietary product, or vendor does not constitute a guarantee or warranty of the product by the United States Department of Agriculture and does not imply its approval to the exclusion of other products or vendors that may also be suitable

Biochemical reagents of the highest purity available were purchased from Sigma–Aldrich (St. Louis, MO, USA). Ribulose 1,5-bisphosphate was synthesized by isomerization and phosphorylation of ribose 5-phosphate (Jordan and Ogren 1984). Rubisco was purified from tobacco or Arabidopsis leaves as described previously and converted to the ER form (Carmo-Silva et al. 2011). Recombinant tobacco and Arabidopsis RCA was expressed in *Escherichia coli* and purified as described previously (van de Loo and Salvucci 1996; Barta et al. 2011).

### Plant material and conditions used for growth

The conditions used for growth of *Arabidopsis thaliana* (L.) Heynh. wild type, cv. Columbia, and the transgenic line rwt43 (Zhang et al. 2002) were described previously (Carmo-Silva and Salvucci 2013). Camelina (*Camelina sativa* (L.) Crantz cv. Robinson) and tobacco (*Nicotiana tabacum* L. cv. Petit Havana) plants, including transgenic tobacco plants that express a His-tagged Rubisco (Rumeau et al. 2004), were grown under the conditions described in Carmo-Silva and Salvucci (2012). Measurements were conducted on fully expanded leaves of 4–5 week old plants of Arabidopsis and camelina, and 5–6 week old plants of tobacco.

### Isolation and expression of cDNAs and protein for dPGM and PEP carboxylase

A cDNA clone for dPGM was isolated from *E. coli* (Fraser et al. 1999) and cloned into pET23a (Novagen, Madison, WI, USA). Nucleotides that encode for a C-terminal Strep-tactin (S-Tag) were added to the cDNA clone by PCR using a modified reverse primer. The modified primer encoded for the eight amino acid S-Tag (W-S-H-P-Q-F-E-K) that was linked to the authentic C-terminus by two amino acids; Ser-Ala. Recombinant dPGM protein containing the S-Tag (dPGM-ST) was expressed in *E coli* BL21Star™(DE3)pLysS as described by van de Loo and Salvucci (1996). Frozen cell pellets containing dPGM-ST were thawed in 0.1 M potassium phosphate, pH 8, containing 75 mM NaCl, (buffer A) and 10 mM 2-mercaptoethanol, 1 mM phenylmethylsulfonyl fluoride and 10 μM leupeptin and sonicated for 3 min at 4 °C. Following centrifugation for 20 min at 26,000×*g*, protein in the extract was precipitated with 80 % ammonium sulphate, collected by centrifugation and suspended in buffer A. Following desalting on a Sephadex G-25 column, the dPGM-ST was purified by passage over a 20 ml column of Strep-tactin Sepharose (IBA GmbH, Goettingen, Germany) that had been equilibrated in buffer A. After washing with 10 column volumes of buffer A, dPGM-ST was eluted with 5 mM desthiobiotin in buffer A. The purified dPGM-ST was precipitated with ammonium sulphate and desalted on a Sephadex G-25 column, equilibrated with 60 mM Tris–HCl, pH 7.9. Fractions containing protein were pooled and stored at −80 °C.

For the initial development of the assay, PEP carboxylase was purified from maize leaves by a procedure described for Rubisco (Carmo-Silva et al. 2011). The protein peak corresponding to PEP carboxylase eluted from the ion-exchange column just prior to that of Rubisco. A commercially available PEP carboxylase (Sigma–Aldrich #C1744) from a microbial source was also used in the assay.

### Measurement of RCA activity using purified proteins

RCA activity was measured as the ability to restore activity to the inactive Rubisco-RuBP (ER) complex (Salvucci et al. 1985). Rubisco activity was measured in reactions containing 100 mM Tricine-NaOH, pH 8, 10 mM MgCl_2_, 10 mM NaHCO_3_, 5 mM DTT, 5 % (w/v) PEG-3350, 1 mM NADH, 0.48 U enolase, 0.75 U dPGM-ST, 0.2 mM 2,3-bisPGA, 2 mM RuBP, 10 mM glucose-6-phosphate, 0.75 U PEP carboxylase, 1 U malic dehydrogenase, 5 mM ATP plus ADP at various ratios, and recombinant RCA and Rubisco at the concentrations indicated in the text. For assays using the commercially available microbial PEP carboxylase, the microbial PEP carboxylase (1 U) was substituted for the maize enzyme and glucose-6-phosphate and PEG-3350 were omitted from the mix. To avoid under-estimating activity and to eliminate long lags in product conversion, the specific activities of the linking enzymes were more than tenfold higher than the maximum activity of Rubisco at the highest concentration used. When tested using sub-saturating and saturating concentrations of 3-PGA, the activities of the linking enzymes catalysed NADH oxidation at rates that were several-fold higher than the maximum rate of Rubisco activity.

Rubisco assays were conducted at 30 °C in 96 well plates in a total volume of 0.1 or 0.2 mL. RCA was added to reactions containing all of the components except Rubisco. After 30 s, reactions were initiated with Rubisco in the ER form and the decrease in absorbance at 340 nm, linked to the stoichiometric production of 3-PGA, was measured continuously using a Synergy HT (Bio-Tek, Denkendorf, Germany) plate reader. To determine the activity of the fully carbamylated ECM form, reactions were first incubated for 3 min without RuBP. When incubated without RuBP, the ER form rapidly carbamylated in the presence of RCA and ATP (Robinson and Portis 1988).

### Measurement of Rubisco activation state

For measurement of Rubisco activation, leaf discs (0.5 cm^2^) were excised from the plants and floated on a solution of 25 mM MES-NaOH, pH 5.5, contained within a water-jacketed beaker. The solution was flushed with humidified air (380 μL L^−1^ CO_2_ in 21 % O_2_, balance N_2_) under the conditions of irradiance and temperature indicated in the text. After each treatment, leaf discs were quickly frozen in liquid nitrogen and stored at −80 °C.

Samples consisting of one or two frozen leaf discs, (0.5–1 cm^2^), were extracted in Ten Broeck glass homogenisers with 1 mL cm^−2^ of 100 mM Tricine-NaOH, pH 8, 5 mM MgCl_2_, 1 mM EDTA, 5 % PVP-40, 6 % PEG-4000, 5 mM DTT, 1 mM phenylmethylsulfonyl fluoride and 10 μM leupeptin. Assays were conducted at 30 °C either immediately after extraction or after centrifugation for 20 s at 10,000×*g*. To measure initial Rubisco activity, 0.02 mL of leaf extract was added to assay mix in clear 96 well plates to a final volume of 0.2 mL. The assay mix contained 100 mM Tricine-NaOH, pH 8, 10 mM MgCl_2_, 10 mM NaHCO_3_, 20 mM KCl, 5 mM DTT, 1 mM NADH, 1.85 U pyruvate kinase, 2.33 U lactate dehydrogenase, 0.96 U enolase, 0.75 U dPGM, 0.2 mM 2,3-bisPGA, 2 mM ADP and 0.5 mM RuBP. To measure total activity, leaf extracts were incubated in the assay mix without RuBP to fully carbamylate Rubisco (Carmo-Silva and Salvucci 2013). The rate of decrease in absorbance at 340 nm during the first 1–2 min of the assay was measured using a Synergy HT (Bio-Tek, Denkendorf, Germany) plate reader immediately after addition of the leaf extract to the assay mix containing 1 mM RuBP (initial), or after 3 min incubation in the assay mix prior to addition of RuBP (total). For some experiments, assays were conducted in microcuvettes and the absorbance at 340 nm was monitored using a UV–Vis spectrophotometer (Varian, Cary Bio100). For these reactions, the total assay volume was 0.4 mL and the leaf extract volume was 0.04 mL.

### Two stage assay for Rubisco activity using purified proteins

A two-stage assay was also used to assay RCA activity. The first stage assay contained 100 mM Tricine-NaOH, pH 8, 10 mM MgCl_2_, 10 mM NaHCO_3_, 2 mM DTT, 5 mM ATP, 5 mM RuBP, 5 % PEG-3350, and 0.1 mg mL^−1^ tobacco RCA in a total volume of 50 μL. Reactions were initiated with 1 mg mL^−1^ tobacco Rubisco. At set time points, 0.01 mL aliquots were transferred to microtubes containing 0.03 mL of 100 mM Tricine-NaOH, pH 8 at 95 °C to stop the reactions. To determine the amount of 3-PGA formed during the first stage, 15 μL aliquots of the quenched samples were added to 185 μL of 100 mM Tricine-NaOH, pH 8, 10 mM MgCl_2_, 10 mM NaHCO_3_, 5 mM DTT, 1 mM NADH, 0.96 U enolase, 0.75 U dPGM, 0.2 mM 2,3-bisPGA, 1.85 U pyruvate kinase, 2.33 U lactate dehydrogenase and 2 mM ADP. The change in absorbance at 340 nm was measured as described above using a plate reader.

### Data analysis

The carboxylase activity of Rubisco, expressed as μmol CO_2_ incorporated min^−1^ mg^−1^ protein or converted to k_cat_ (s^−1^), i.e., turnover number, was determined from the stoichiometric production of two molecules of 3-PGA per molecule of CO_2_ fixed. The rate of 3-PGA production was determined continuously from the decrease in absorbance at 340 nm due to the oxidation of NADH and converted to Rubisco specific activity. To determine the fraction of sites activated, the specific activity was divided by the specific activity of the fully carbamylated Rubisco, i.e., ECM = 100 % of the sites carbamylated.

RCA affects both the rate and the final extent of Rubisco activation (van de Loo and Salvucci 1996). Consequently, for experiments comparing different RCAs or Rubiscos, RCA activity was based on the final steady-state specific activity of Rubisco and then converted to the fraction of Rubisco sites activated after interacting with RCA. To determine the effect of RCA and Rubisco concentrations on the rate of Rubisco activation, the fraction of Rubisco sites activated min^−1^ was determined from a linear regression of the progress curve at each concentration of RCA and Rubisco. Adjusting the rate for the amounts of RCA and Rubisco made it possible to calculate the specific activity of RCA as mol Rubisco sites activated min^−1^ mol^−1^ RCA protomer.

All assays were conducted in at least triplicate and the results are the mean ± SE. Statistical comparisons between different treatments were made using analysis of variance (ANOVA) followed by the Holm-Sidak method for multiple pairwise comparisons (for more than two treatments). *P*-values lower than 0.05 were considered statistically significant.

### Miscellaneous

Protein concentration in leaf extracts was determined by the method of Bradford (1976). The same method was used to determine the concentration of RCA protein. Rubisco protein was determined based on the extinction coefficient at 280 nm (Paulsen and Lane 1966).

## Results

### Considerations in developing the assay

The most important consideration in developing a continuous assay for RCA was the requirement for analysing the main regulatory property of the enzyme, i.e., the response of activity to variable ratios of ADP:ATP. To satisfy this criterion, a method was devised for coupling 3-PGA formation to pyridine nucleotide oxidation that was independent of adenine nucleotides. The method involved converting 3-PGA to PEP using dPGM and enolase and then coupling PEP production to the oxidation of NADH using PEP carboxylase and malic dehydrogenase (Fig. 1a).

For the first step, 2,3-bisPGA-dPGM was selected over the cofactor-independent PGM because of its higher specific activity and lower affinity for 2-PGA (Fraser et al. 1999). To our knowledge, dPGM is not commercially available but the cDNA that encodes for the protein can be isolated from and expressed in *E. coli*. By using a pET expression system similar to the one described previously (Fraser et al. 1999), and including a C-terminal S-tag to facilitate purification, copious amounts of soluble dPGM-ST protein could be isolated in recombinant form. The additional eight amino acids of the S-tag plus a two amino acid linker did not interfere with expression or activity (data not shown).

Conversion of 3-PGA via dPGM and enolase produces PEP that was then linked to NADH oxidation using PEP carboxylase and malate dehydrogenase. Formation of PEP can also be linked to NADH oxidation via pyruvate kinase and lactate dehydrogenase. However, this link cannot be used for continuous measurement of RCA activity because the pyruvate kinase reaction requires ADP, an inhibitor of RCA (see below). For the initial experiments using PEP carboxylase, the enzyme was purified from maize leaves. Active maize PEP carboxylase with an N-terminal affinity tag has been expressed in recombinant form (Dong et al. 1997). Thus, the recombinant maize enzyme could be used as a ready source of PEP carboxylase for the RCA assay. In addition, a relatively inexpensive microbial PEP carboxylase is available commercially. This enzyme exhibited very low activity in the standard assay due to precipitation of the protein by PEG. By removing PEG from the assay mix, the commercially available microbial PEP carboxylase was a suitable substitute for maize PEP carboxylase in the RCA assay.

In preliminary experiments, the oxidation of NADH in the coupled system using maize PEP carboxylase was very slow when the concentration of 3-PGA was low, even though the activities of the coupling enzymes were in excess based on their specific activities at saturating substrate concentrations. Addition of the PEP carboxylase activator, glucose-6-phosphate, to the assay greatly increased the rates, indicating that the assay system required this effector to overcome the low affinity of maize PEP carboxylase for PEP (Coombs et al. 1973). In contrast, the activity of the microbial PEP carboxylase was unaffected by glucose-6-phosphate, catalysing the linked reaction at adequate rates for the assay of Rubisco.

### Validation of the assay I: effect of Rubisco and RCA concentration on RCA activity

**Fig. 2:**
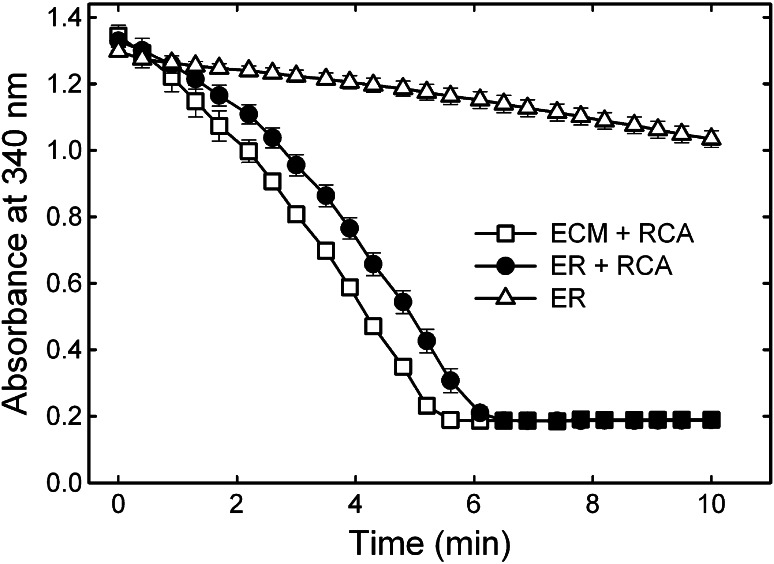
Continuous measurement of Rubisco activity demonstrating the conversion of Rubisco from the inactive ER to the active ECM form by RCA. The data show the time course of the decrease in A_340_ in assays linking RuBP-dependent 3-PGA formation to NADH oxidation (see Fig. 1a). Reactions contained either 0.1 mg mL^−1^ tobacco Rubisco in the fully carbamylated ECM form plus 0.1 mg mL^−1^ tobacco RCA (*open squares*), 0.1 mg mL^−1^ tobacco Rubisco in the ER form (*open triangles*) or 0.1 mg mL^−1^ tobacco Rubisco in the ER form plus 0.1 mg mL^−1^ tobacco RCA (*closed circles*). All reactions were conducted at 30 °C and contained 5 mM ATP

RCA activity can be measured by its ability to increase the activity of uncarbamylated Rubisco containing tightly-bound RuBP, commonly referred to as the ER form of the enzyme. This form of Rubisco is inactive and slow to activate, in contrast to the active ECM form that is fully carbamylated and contains bound Mg^2+^. As shown in Fig. 2, the dPGM-based assay developed here was suitable for measuring the activity of Rubisco, as evidenced by the marked differences in the rate of NADH oxidation between the ER and ECM forms of Rubisco. Similarly, the increased rate of NADH oxidation from the conversion of the inactive ER to the active ECM form of Rubisco was apparent when ER was added to reactions containing ATP and RCA. The raw data show that with RCA, Rubisco activity increases progressively during the time course, indicating that the proportion of Rubisco in the active form increases with time.

**Fig. 3:**
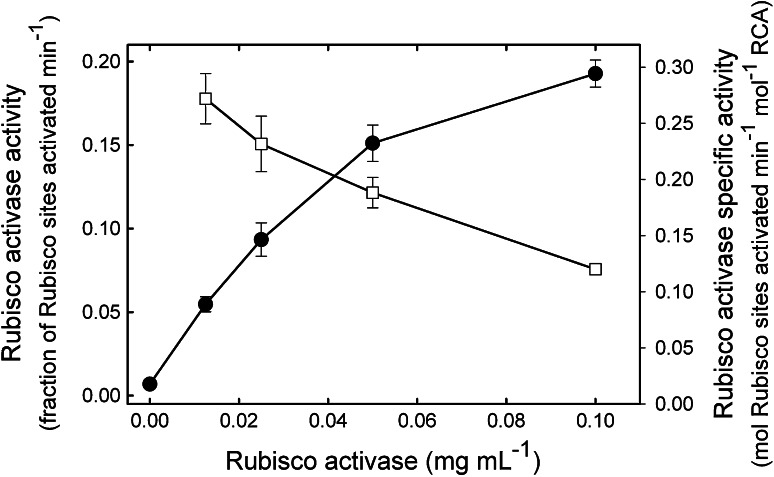
Effect of RCA concentration on RCA activity. Tobacco Rubisco in the ER form was incubated with the indicated concentrations of tobacco RCA at 30 °C in the presence of 5 mM ATP. Rubisco activity was measured continuously as described in Fig. 2 and the fraction of sites activated was determined at each time point. From a linear regression of the progress curve, RCA activity was determined at each concentration of RCA as the fraction of Rubisco sites activated min^−1^ (*filled circle*). The specific activity of RCA, mol Rubisco sites activated min^−1^ mol^−1^ RCA protomer (*open squares*), was calculated using these rates and the amounts of Rubisco and RCA protein in the assays

To demonstrate the versatility of the assay, the dependence of RCA and Rubisco concentrations on activation of the inactive ER complex by RCA was examined (Fig. 3, Supplemental Fig. S1). As shown previously using the timed, two-stage ^14^C assay (Robinson and Portis 1988), the rate of activation of Rubisco, measured as the fraction of Rubisco sites activated per min, increased with increasing concentrations of RCA. However, the specific activity of RCA, i.e., mol Rubisco sites activated min^−1^ mol^−1^ RCA protomer, decreased with increasing RCA concentration (Fig. 3). These results indicate that, at the concentrations of Rubisco and RCA protein used here, the rate of Rubisco activation per mol of RCA protein decreased with increasing ratios of RCA to Rubisco. In contrast, at a constant concentration of RCA, the specific activity of RCA increased with increasing amounts of ER (Supplemental Fig. S1).

### Validation of the assay II: effect of ADP/ATP on RCA activity

**Fig. 4:**
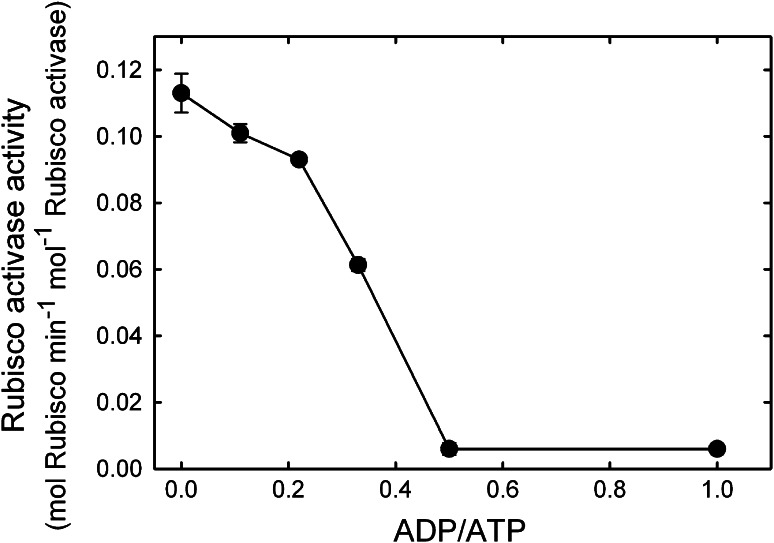
Effect of ADP:ATP ratio on the activity of RCA. Tobacco Rubisco at 0.1 mg mL^−1^ was incubated in the ER form with 0.1 mg mL^−1^ tobacco RCA at 30 °C in the presence of 5 mM ATP plus ATP, at the indicated ratios. Rubisco activity was measured continuously as described in Fig. 2 and the fraction of sites activated was determined at each time point. From a linear regression of the progress curve, RCA activity was determined at each ratio of ADP:ATP as the fraction of Rubisco sites activated min^−1^ and converted to RCA specific activity, mol Rubisco sites activated min^−1^ mol^−1^ RCA protomer (*filled circle*), by adjusting the rate for the amounts of Rubisco and RCA protein in the assays

To further validate the continuous assay system (Fig. 1a) the effect of ADP: ATP ratio on RCA activity was investigated (Fig. 4). As shown previously, RCA activity decreased as the ratio of ADP:ATP increased. At a ratio of 0.5, the activity of ER in the presence of RCA and ATP was not statistically different from the activity determined without RCA, indicating that tobacco RCA was completely inactive. With physiological ratios of 0.33 ADP: ATP (Stitt et al. 1982; Zhang and Portis 1999) the rate of Rubisco activation by RCA was reduced by 46 % compared to the rate with no ADP.

In a separate set of experiments, the effect of ADP on RCA activity was compared for the β-isoforms of RCA from tobacco and Arabidopsis (Supplemental Table S1). Previous studies using the ^14^C Rubisco assay have shown that the β-RCA from Arabidopsis is much less inhibited by ADP than the enzyme from tobacco (Carmo-Silva and Salvucci 2013). Measurements using the continuous assay confirmed these findings; at 0.33 ADP:ATP the Arabidopsis β-RCA was inhibited by 25 % compared with 65 % inhibition of the tobacco enzyme.

### Validation of the assay III: measuring activation of polyhistidine-modified Rubisco by RCA

**Fig. 5:**
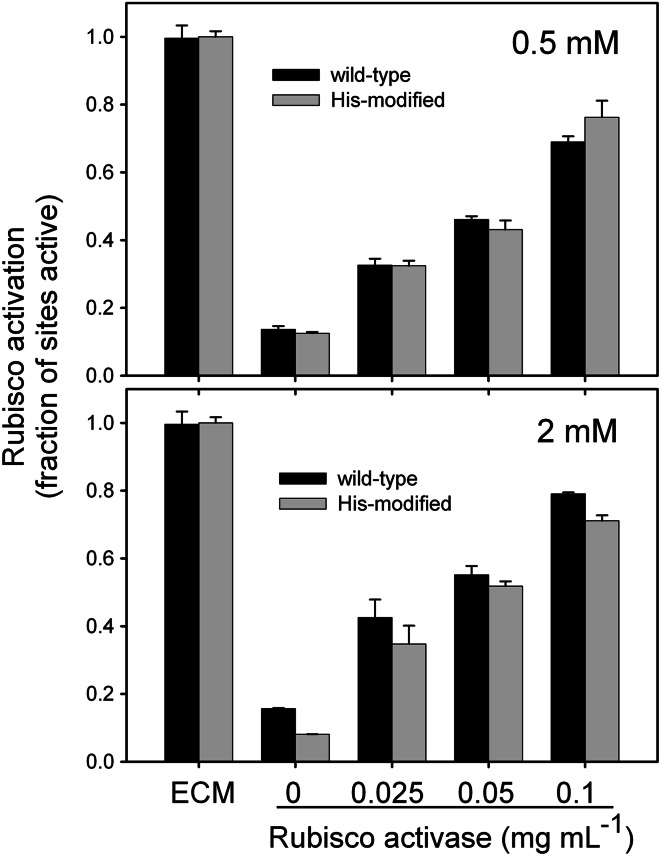
Activation of wild-type and His-tagged modified Rubisco by RCA. Tobacco Rubisco at 0.1 mg mL^−1^ was incubated in the ER form with the indicated amounts of tobacco RCA at 30 °C in the presence of 5 mM ATP or converted to ECM form by incubation with CO_2_ and Mg^2+^. Assays were completed with either 0.5 mM or 2 mM RuBP. Rubisco activity was measured continuously as described in Fig. 2 and the fraction of sites activated was determined by comparing the steady-state activity with the activity of the fully carbamylated enzyme (ECM)

In another test of the assay, the continuous assay for RCA activity was used to determine if the addition of six histidine residues to the C-terminus of the large subunit of Rubisco (Rumeau et al. 2004) affected Rubisco activity or activation of Rubisco by RCA (Fig. 5). Measurement of the specific activities of the ECM form of wild-type and modified Rubisco, 0.83 ± 0.03 and 0.78 ± 0.01 U mg^−1^ protein, respectively, indicated that the poly-His addition did not significantly affect the maximal carboxylase activity. Similarly, the activity of the ER forms of both of these enzymes remained below 20 % of the maximum when incubated with high CO_2_ and Mg^2+^ in the presence of 0.5 and 2 mM RuBP. The low activity of the His-modified Rubisco indicated that the stability of the ER complex was not markedly affected by the modification. Finally, the extent of activation of the ER form of the polyhistidine-modified Rubisco by various amounts of tobacco RCA was similar to wild-type Rubisco at both 0.5 and 2 mM RuBP. These results indicate that the effectiveness of RCA in converting Rubisco from the inactive ER form to the active ECM form was not compromised by extending the C-terminus of the large subunit of Rubisco by six histidine residues.

### Modification of the standard assay for measuring Rubisco activity and activation

The activation state of Rubisco in leaves can be determined by measuring the activity of Rubisco in rapidly prepared leaf extracts, i.e., initial activity, and comparing this activity to the activity of the fully carbamylated enzyme, i.e. total activity (Perchorowicz et al. 1981). To measure Rubisco activation, the standard assay described above (Fig. 1a) or a modified version (Fig. 1b) can be used as a stand-alone assay for either purified Rubisco or Rubisco in leaf extracts (Fig. 1b). The modified version still uses dPGM-ST and enolase to convert 3-PGA to PEP, but couples PEP formation to NADH oxidation via pyruvate kinase and lactate dehydrogenase. The pyruvate kinase-lactate dehydrogenase link requires ADP, a potent inhibitor of RCA, but not Rubisco. Thus, these linking enzymes, while suitable for measuring Rubisco activity per se, cannot be used for measuring the effects of RCA on Rubisco activity in a continuous assay. The main advantage of the modified assay for measuring Rubisco is that the two linking enzymes are commercially available and inexpensive.

To demonstrate the usefulness of the assay for measuring Rubisco activation, the effect of irradiance on the activation state of Rubisco was determined in wild-type and transgenic Arabidopsis using the modified assay (Fig. 1b). As shown in Table 1, the results demonstrated that the assay was capable of measuring light-dependent changes in Rubisco activation that occur in wild-type plants. The measurements also confirmed that (1) deactivation of Rubisco in response to low light was minimal in the rwt43 transformant, a transgenic Arabidopsis that expresses only the ADP-insensitive β-isoform of RCA (Carmo-Silva and Salvucci 2013) and (2) the rates of Rubisco activity in crude leaf extracts of wild-type and transgenic plants were similar to those determined with a ^14^C-based Rubisco assay (Salvucci et al. 2006). In a separate set of experiments, the non-radioactive assay was used to detect the decrease in Rubisco activation state that occurred in camelina plants subjected to heat stress (Supplemental Table S2). These results confirmed previous findings obtained using the ^14^C assay (Carmo-Silva and Salvucci 2012).

**Table 1:** Effect of irradiance on the activation state of Rubisco in wild type Arabidopsis and the transgenic line, rwt43

Arabidopsis line	Irradiance (μE m^−2^ s^−1^)	Rubisco activity		Activation (%)
		Initial	Total	
		(μmol min^−1^ mg^−1^ prot)		
Wild type	1200	0.40 ± 0.03	0.46 ± 0.08	86 ± 3^a^
	75	0.35 ± 0.03	0.59 ± 0.02	60 ± 4^b^
	25	0.13 ± 0.01	0.59 ± 0.03	23 ± 2^c^
rwt43	1200	0.42 ± 0.08	0.45 ± 0.07	91 ± 4^a^
	75	0.45 ± 0.04	0.53 ± 0.04	85 ± 4^a^
	25	0.47 ± 0.03	0.55 ± 0.04	87 ± 3^a^

Leaf discs were exposed to the indicated irradiance for 120 min prior to sampling. Letters indicate activation states that are statistically different at the *P* = <0.001 level

### Two-stage assay for high-throughput analysis

While continuous assays can be automated for high-throughput analysis, it is often more convenient to conduct two-stage, timed assays and then allow product conversion in the second stage to run to completion (Gibon et al. 2004). Either the standard or modified assay described above could be used as a two-stage assay. To demonstrate this fact, RCA activity was measured in two stages using a timed assay to determine the suitability of the dPGM-linked reaction sequence for automation. In the first stage, Rubisco in the ER form was incubated with RuBP, ATP and RCA before heating at 95 °C. The 3-PGA produced during the first stage was then determined by adding an aliquot of the reaction to a second stage assay that converted 3-PGA to lactic acid (Fig. 1b). The data showed that it was possible to measure activation of the ER form of Rubisco by RCA using this two-stage assay with a single time point (Supplemental Table S3).

## Discussion

### The interaction of Rubisco and RCA

The physical interaction between RCA and Rubisco has long been enigmatic, presumably because of the transient nature of the binary complex. Rubisco and RCA do not form a stable binary complex that would facilitate a thorough characterisation of the molecular details of the interaction (Portis et al. 2008; Blayney et al. 2011). However, the consequence of the interaction can easily be detected by measuring the effect of RCA on Rubisco activity (Salvucci et al. 1985). In the presence of ATP, RCA increases the activity of inhibited forms of Rubisco, i.e., forms produced by the tight binding of certain sugar-phosphates (Portis 2003), including the unproductive binding of the substrate, RuBP, to uncarbamylated enzyme. Wang and Portis (1992) showed that the increases in Rubisco activity that resulted from the productive interaction of ER with RCA were associated with more rapid dissociation of inhibitory sugar-phosphates. These data indicate that “activation of Rubisco” by RCA involves altering the positions of specific domains around the Rubisco active site to allow bound sugar-phosphates to dissociate more rapidly. Although the precise nature of the interaction between RCA and Rubisco is unknown, specific residues of both Rubisco and RCA that are involved in the interaction have been identified (Ott et al. 2000; Larson et al. 1997; Li et al. 2005; Portis et al. 2008). The positions of these residues suggest some possibilities for how RCA remodels the conformation of Rubisco (Stotz et al. 2011; Henderson et al. 2011; Wachter et al. 2013).

### Significance of measuring RCA activity at variable ratios of ADP:ATP

The effect of RCA on Rubisco activity has been investigated most often using purified proteins in a simple, timed assay that measures the incorporation of radioactive carbon from CO_2_ into acid-stable products. A high throughput version of this assay was even used to screen for RCA variants with increased thermotolerance (Kurek et al. 2007). However, the logistical issues associated with using radioactive material provide motivation for developing a versatile, non-radioactive assay that could be used for measuring Rubisco and RCA activity. With modifications, the basic assay could also be used as an inexpensive method for measuring the activation state of Rubisco.

Unlike other photometric assays (Sharkey et al. 1991; Sulpice et al. 2007), the continuous assay described here could be used to measure the activity of RCA in the presence of variable ratios of ADP:ATP. This feature is an important consideration since the ratio of ADP:ATP is a major factor regulating the activity of RCA in plants (Robinson and Portis 1989a) and influencing the rate of photosynthetic induction (Carmo-Silva and Salvucci 2013). This fact was demonstrated in studies using Arabidopsis plants that express forms of RCA that differ in their sensitivity to ADP. These plants exhibit marked differences in the response of Rubisco activation to irradiance (Zhang et al. 2002; Carmo-Silva and Salvucci 2013). As a result, plants whose RCA was less sensitive to inhibition by ADP exhibited faster rates of photosynthetic induction during transitions from low to high irradiance because Rubisco was already highly active under low irradiance in these plants (Carmo-Silva and Salvucci 2013, see also Table 1). This finding indicates that manipulating the regulatory properties of RCA might provide a strategy for increasing the rate of photosynthesis in variable light environments.

The assay described here should provide a useful tool for evaluating the interaction between Rubisco and RCA, including variants of both proteins. To demonstrate this application, the activation of a His-tagged Rubisco by RCA was measured to test the hypothesis that RCA alters the conformation of Rubisco via a pore threading mechanism involving movement of the C-terminus of the Rubisco large subunit by RCA (Mueller-Cajar et al. 2011; Stotz et al. 2011). While the data did not conclusively support or reject the hypothesis, they show that the interaction of RCA with Rubisco is unaffected by extending the C-terminus of the large subunit of Rubisco by six histidine residues.

### Measuring Rubisco activity and Rubisco activation state

Due to the investment associated with producing the dPGM-ST used in the RCA assay, it was desirable to use the central portion of the assay, the conversion of 3-PGA to PEP, to measure Rubisco activation in leaf extracts. These assays demonstrated the influence of both irradiance and temperature on the activation state of Rubisco in leaves, verifying that the amount of active Rubisco changes in response to these environmental factors.

The high sensitivity of ^14^C-based assays for Rubisco allow for very short reaction times, i.e. 30–60 s (Lorimer et al. 1977). Short reaction times minimize the problem with “fall-over”; the slow, progressive decrease in catalytic activity caused by either the presence of inhibitory compounds in the RuBP preparation (Kane et al. 1998) or the production of catalytic misfire products at the active-site (Edmondson et al. 1990). It should be noted that fall-over does not occur in assays containing active RCA, because RCA reverses the tight-binding of the inhibitory sugar-phosphates (Robinson and Portis 1989b). However, a fall-over type decline occurred during the later time points (i.e., after 5–10 min) in assays of Rubisco that did not contain RCA (data not shown). For this reason, we recommend determining Rubisco activity and Rubisco activation during the initial 1–2 min when the activity decline is negligible (Robinson and Portis 1989b).

## Summary

The continuous photometric assay described here for measuring the activities of Rubisco and RCA is flexible and easily adaptable to a variety of experimental situations, including for use with purified proteins and leaf extracts. All but one of the linking enzymes is commercially available and the dPGM-ST can be produced in *E. coli* and isolated by affinity chromatography. The assays can be conducted in microplates and the changes in absorbance detected using a plate reader. The basic assay for RCA activity described in Fig. 1a could be prepared as a master mix containing all of the components except Rubisco, RCA and RuBP. The master mix was stable when stored either frozen at −80 °C or lyophilized at 4 °C. By dividing the assay into two stages, the assay can be used in a high-throughput or robotic system. While the assay described here provides a reliable measurement of the carboxylase activity of Rubisco, the simultaneous assay of carboxylase and oxygenase activity using ^14^CO_2_ and ^3^H-RuBP developed by Jordan and Ogren (1981) is still the most accurate method for determining the substrate specificity of Rubisco. With a growing interest in Rubisco regulation, the assay described here provides a timely alternative to radioactive assays for measuring Rubisco and RCA activity.

## Electronic supplementary material

Below is the link to the electronic supplementary material.

Supplementary material 1 (DOCX 977 kb)

## Abbreviations

2,3-bisPGA: 2,3-Bisphosphoglycerate
dPGM: 2,3-Bisphosphate-dependent phosphoglycerate mutase
dPGM-ST: 2,3-Bisphosphate-dependent phosphoglycerate mutase containing a Strep-tactin binding peptide
ER: Rubisco complexed with RuBP
ECM: Rubisco in the carbamylated state with a bound metal cation
PEP: Phosphoenolpyruvic acid
3-PGA: 3-Phosphoglyceric acid
RCA: Rubisco activase
RuBP: Ribulose-1,5-bisphosphate
S-Tag: Strep-tactin-binding peptide
