# Supplementary material for: A non-radioactive method for measuring Rubisco activase activity in the presence of variable ATP: ADP ratios, including modifications for measuring the activity and activation state of Rubisco
Source: Photosynth Res. 2014 Jan 5;119(3):355–65. doi: 10.1007/s11120-013-9964-5 (PMC3923112; doi:10.1007/s11120-013-9964-5)
Supplement: Supplementary file 1 — Supplementary material 1 (DOCX 977 kb) [file 11120_2013_9964_MOESM1_ESM.docx]

**Supplementary Material**

**A non-radioactive method for measuring Rubisco activase activity in the presence of variable ATP: ADP ratios, including modifications for measuring the activity and activation state of Rubisco**

**Joanna C. Scales, Martin A. J. Parry and Michael E. Salvucci**

The online version of this article contains supplementary material, which is available to authorized users.

**Supplemental Table S1.** Differences in the sensitivity of β-isoform Rubisco activases to inhibition by ADP.

**Supplemental Table S2.** Effect of temperature on the activation state of Rubisco in camelina

**Supplemental Table S3.** Effect of Rubisco activase on the activation of Rubisco, measured in a two-stage assay.

**Supplemental Figure S1.** Effect of Rubisco concentration on Rubisco activase (RCA) activity

**Supplemental Table S1.** Differences in the sensitivity of β-isoform Rubisco activases (RCA) to inhibition by ADP. Recombinant RCA from tobacco and Arabidopsis were incubated at 0.1 mg mL^-1^ with 0.075 mg mL^-1^ of the ER forms of tobacco and Arabidopsis Rubisco, respectively, in the presence of either 5 mM ATP or 5 mM ATP plus ADP at a ratio of 0.33 ADP:ATP. Rubisco activity was measured continuously using the assay shown in Fig. 1A. Maximum rates were determined and used to calculate k_cat_. The values in parentheses indicate the fraction of Rubisco sites that were activated. The turnover rates of the ER forms in the absence of RCA were 0.08 and 0.2 s^-1^ for tobacco and Arabidopsis Rubisco, respectively.

| Plant species | Rubisco activity (k_cat_) | |
| --- | --- | --- |
|  | ADP/ATP | |
|  | 0 | 0.33 |
| tobacco | 1.73 ± 0.05 (100) | 0.53 ± 0.05 (0.35) |
| Arabidopsis | 2.96 ± 0.06 (0.95) | 2.21 ± 0.06 (0.71) |

**Supplemental Table S2.** Effect of temperature on the activation state of Rubisco in camelina. Letters indicate activation states that are statistically different at the P=<0.001 level.

|  | Rubisco activity | |  |
| --- | --- | --- | --- |
| Temperature | Initial | Total | Activation |
| (°C) | (µmol min^-1^ mg^-1^ protein) | | (%) |
| 23 | 0.34 ± 0.01 | 0.40 ± 0.00 | 84 ± 0.5^a^ |
| 42 | 0.18 ± 0.01 | 0.32 ± 0.01 | 55 ± 1.0^b^ |

**Supplemental Table S3.** Effect of Rubisco activase (RCA) on the activation of Rubisco, measured in a two-stage assay. Rubisco activity was determined by the amount of 3-PGA formed after 5 min in reactions containing 1 mg mL^-1^ of the ER form of tobacco Rubisco in the presence and absence of tobacco RCA. The activity of the ECM form of Rubisco was measured by incubating the Rubisco with 10 mM MgCl_2_ and NaHCO_3_ in the absence of RuBP for 10 min prior to assay.

|  | Rubisco activity | |
| --- | --- | --- |
| Rubisco form | RCA  (mg mL^-1^) | Rubisco activation  (fraction of sites active) |
| ER | 0 | 0.12 ± 0.01 |
| ER | 0.1 | 0.71 ± 0.03 |
| ECM | 0.1 | 1.00 ± 0.06 |


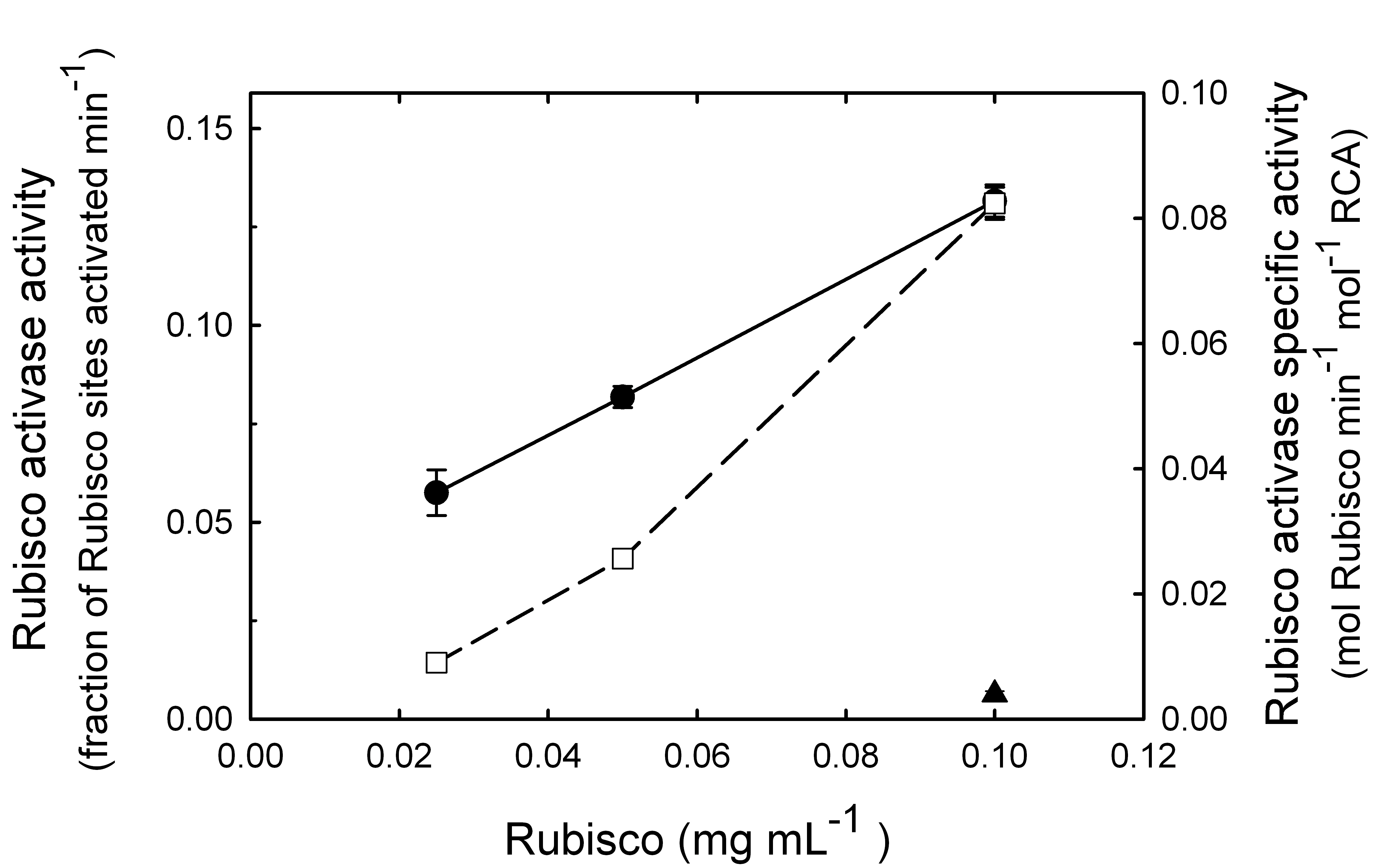


**Supplemental Figure S1.** Effect of Rubisco concentration on Rubisco activase (RCA) activity. Tobacco RCA at 0.1 mg ml^-1^ was incubated with the indicated concentrations of tobacco Rubisco in the ER form and Rubisco activity at 30°C in the presence of 5 mM ATP. Rubisco activity was measured continuously as described in Fig. 2 and the fraction of sites activated was determined at each time point. From a linear regression of the progress curve, RCA activity was determined for each concentration of Rubisco as the fraction of Rubisco sites activated min^-1^ (filled circle). The rate of spontaneous activation of the ER form in the absence of RCA is indicated by the closed triangle. The specific activity of RCA, mol Rubisco sites activated min^-1^ mol^-1^ RCA protomer (open squares), was calculated by adjusting the rate for the amounts of Rubisco and RCA in the assays.
